# Single-center experience and evaluation of rare ıntracranial abscesses in childhood

**DOI:** 10.1186/s13052-025-01895-y

**Published:** 2025-02-07

**Authors:** Nihal Yildiz, Zeynep Gökçe Gayretli Aydin

**Affiliations:** 1 Faculty of Medicine, Department of Pediatric Neurology, Zonguldak Bülent Ecevit University, Zonguldak, Turkey; 2 Faculty of Medicine, Department of Pediatric Infection, Farabi Hospital, Karadeniz Technical Unıversity, Trabzon, Turkey

**Keywords:** Brain absesses, Brain infectious diseases, Childhood, Pediatric brain abscesses, Demographics, Etiology, Treatment, Challenges, Research

## Abstract

**Background:**

Intracranial abscess (IA) is a rare disorder in childhood. Clinical manifestations of brain abscess include headache, fever, and focal neurological deficits. This study aimed to examine the demographic, clinical, laboratory, and imaging findings in children with IA.

**Methods:**

Children admitted to the pediatric infection service with a diagnosis of IA between 2011 and 2022 were included in the study. Abscesses were divided into two groups: infratentorial and supratentorial. Demographic characteristics of the patients, complaints, MRI findings, and follow-up data were recorded and compared between the two groups.

**Results:**

The study included a total of 23 patients, 9 (39.1%) of whom were male, with a mean age at diagnosis of 79.3 ± 65.4 months. The most common complaints were headache (39.1%), fever (91.3%), focal neurological deficits (60.9%), seizures, loss of consciousness (26.1%), and meningitis findings (60.9%). The most frequent etiology was post-operative procedures (30.4%), followed by a history of meningomyelocele (13%), and congenital heart disease (8.7%). On MRI, 68.7% of the supratentorial abscesses were multiple and commonly localized in the frontal and parietal regions. Treatment included ceftriaxone (82.6%), vancomycin (65.2%), meropenem (43.5%), metronidazole (34.8%), and linezolid (17.4%). The median hospitalization duration for patients was 32 days (range: 14–150). Of the patients, 34.8% were hospitalized and followed in the intensive care unit, and neurosurgery performed surgical interventions in 60.9% of cases, with evacuation in 21.7% of cases. In cultures, the causative agent was identified on average within 4 ± 1.3 days. Recurrence of abscess occurred in three (13%) cases, and 13% of cases had residual sequelae.

**Conclusions:**

Intracranial abscess is a rare infectious disease that can result in long-term neurological deficits requiring extended follow-up and treatment. A correct and effective approach also positively impacts the prognosis of patients.

## Background

 A brain abscess is a focal infectious formation that can develop within the brain parenchyma, either as a complication of another infection or as a result of trauma or surgery [1]. The tissue damage observed in a brain abscess primarily stems from the acute inflammatory response of the host to potential pathogens [2]. Case reports and broader retrospective studies encompass a variety of organisms as the cause of brain abscess [3–7]. The most common causative agents of brain abscesses are Streptococcus spp. and Staphylococcus spp. strains. However, brain abscesses are rare in childhood, with an estimated incidence of less than 1 per 100,000 [1].

Clinical signs of a brain abscess typically include headache, fever, and focal neurological deficits. Nevertheless, in the early stages of the disease, symptoms may be nonspecific [8]. Patients suspected of having a brain abscess should undergo diagnostic imaging, with magnetic resonance imaging (MRI) being the preferred imaging modality due to its higher sensitivity compared to computed tomography (CT) scanning [9].

In this study, we aimed to examine the demographic, clinical, laboratory, and imaging findings of cases undergoing follow-up for brain abscesses.

## Materials and methods

In this retrospective study, we included children aged between 1 and 18 years who were under the care of the pediatric infection service with a diagnosis of abscess between 2011 and 2022. Patients were categorized into two groups: group-1 (A) comprised those with supratentorial abscesses, while group-2 (B) included patients with infratentorial abscesses.

We collected demographic information on the patients, their symptoms upon admission, MRI findings, laboratory data, and details of the treatments administered from the patients’ medical records.

### Study design

This study was registered at the Karadeniz Technical University Faculty of Medicine Ethical Committee in accordance with the Declaration of Helsinki (Clinical Trial Number: 24237859-830).

### Statistical analysis

Statistical analysis was performed using SPSS 26.0. Descriptive statistics were used for the evaluation results, including numbers, percentages for categorical variables, and mean, standard deviation, median, and minimum-maximum for numerical variables. To assess the normal distribution of measured variables, we employed the Kolmogorov-Smirnov and Shapiro-Wilk tests. Variables conforming to a normal distribution were compared using the t-test or ANOVA, while those not fitting the normal distribution were compared using the Mann-Whitney U Test or Kruskall Wallis Analysis of Variance. For the analysis of categorical data, we employed the Pearson chi-square test or Fisher’s Exact Test. A significance level of *p* < 0.05 was considered for all statistical analyses.

## Results

A total of 23 patients were included in the study, with 9 (39.1%) being male. The mean age at the time of diagnosis was 79.3 ± 65.4 months [In group-1(A) 77 (7–192) ; in group-2 (B) 31 (2–152); *p*=,087]. A had a majority of female patients, while B had a dominance of male patients. The median length of hospitalization was 32 (14–150) days in A and 26,50 (15–45) days in B. The time interval between symptom onset and hospital admission was 10 [2–14, 15] days in A and 3,50 [1–6, 7] days in B, and it was statistically significant (*p* =,006).

No statistically significant differences were observed in terms of perinatal characteristics, such as prematurity, esophageal atresia, congenital heart disease, anal atresia, meningomyelocele, or growth retardation, between the two groups. The most common symptoms observed in the patients were fever (91.3%), focal neurological deficits (60.9%), concomitant meningitis findings (60.9%), headache (39.1%), seizures, and loss of consciousness (26.1%), as shown in Table 1.

**Table 1:** The signs and symptoms of patients at administiration

Symptom	*n* (%)
Fever	%91,3
Focal neurological defect	%60,9
Concomitant meningitis	%60,9
Nausea, vomiting	%52,2
Headache	%39,1
Increased intracranial pressure	%34,8
Seizure	%26,1
Neck stiffness	%26,1
Loss of consciousness	%26,1
Speech disorder	%21,7
Papilledema	%13
Backache	%13
Diplopia	%8,7

**Fig. 1:**
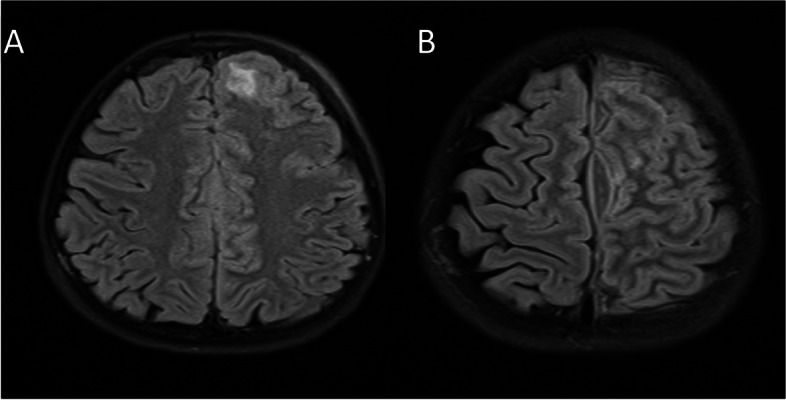
A frontal abscess with cytotoxic edema. Subdural empyema in the extraaxial area adjacent to the falx cerebri at the vertex level and in the left frontoparietal convexity, cytotoxic edema in the left frontal lobe, collection area consistent with an abscess extending from the left orbital superior wall to the subcutaneous tissue, paranasal sinusitis

**Fig. 2:**
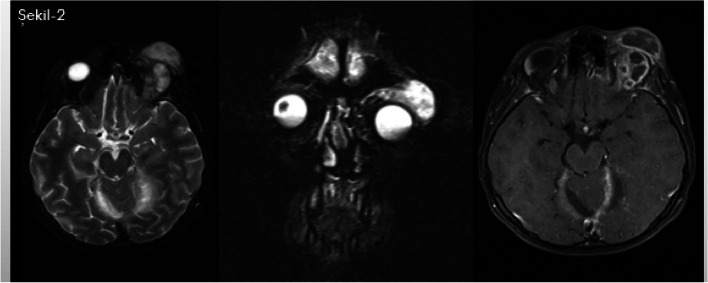
Left preseptal and postseptal extraconal abscess formations, subdural empyemas

The etiology of abscess formation was carefully examined. The most frequent causes were post-operative neurological procedures and otopharyngeal infections (30.4%), paranasal sinusitis (17.3%), community-acquired infections (13%), head trauma, and congenital heart disease (4.3%), respectively, shown in Table 2. Periodontal infection was not detected in any of the cases. On MRI, 68.7% of the 69.6% supratentorial abscesses were multiple and primarily localized in the frontal (43.4%) and parietal regions. Details of abscesses seen in the frontal region are provided in Figs. 1 and 2.

**Table 2:** Etiological causes of abscesses

Etiological cause	*n* (%)
Post-operative neurological procedures	7 (%30,4)
Otopharyngeal infections	6 (%26,1)
Paranasal sinusitis	4 (%17,4)
Community-acquired infections	3 (%13)
Head trauma	1 (%4,3)
Congenital heart disease	1 (%4,3)

While the microbiological etiology of absesses were shown in Table 3 and the most detected agent was the Streptococcal strain. The treatments administered included ceftriaxone (82.6%), vancomycin (65.2%), meropenem (43.5%), metronidazole (34.8%), and linezolid (17.4%). The median duration of hospitalization for the patients was 32 (range: 14–150) days. Approximately 34.8% of the patients required hospitalization and follow-up in the intensive care unit, and neurosurgery intervention was necessary in 60.9% of cases, with evacuation performed in 21.7% of cases. In the cultures, the causative agent was identified on average within 4 ± 1.3 days. It was noted that abscess recurrence occurred in three cases (13%), and sequelae persisted in 13% of cases.

**Table 3:** The microbiological etiology of absesses

The microbiological agent	*n* (%)
Staphylococcus epidermis	1 (% 4,3)
Streptococcus constellatus	3 (%13)
Enterobacter aerogenes	1 (% 4,3)
Stenotrophomonas maltophilia	1 (% 4,3)
Bacillus thermoamylovorans	1 (% 4,3)
Enterococcus faecalis	1 (% 4,3)
Acinetobacter baumannii	1 (% 4,3)
Streptococcus intermedius	1 (% 4,3)
Prevotella oris	1 (% 4,3)
Parvimonas micra	2 (% 8,7)
Gemella morbillorum	1 (% 4,3)
Bacteroides fragilis	1 (% 4,3)
Escherichia coli	1 (% 4,3)
Tissierella praeacuta	1 (% 4,3)

## Discussion

This comprehensive investigation into pediatric brain abscesses yields valuable insights into the intricate dynamics of this complex condition, drawing from a diverse array of clinical, demographic, and etiological parameters. The observed gender distribution, with a prevalence of female patients in group A and male patients in group B, introduces an intriguing dimension to our understanding of pediatric brain abscesses. This finding aligns with previous studies, highlighting gender-specific patterns in the epidemiology and outcomes of neurological infections [1]. Further exploration is warranted to discern the potential implications of these gender disparities on disease progression and prognosis.

While the mean age at diagnosis did not reveal significant differences between the two groups, the wide age range emphasizes the heterogeneity within the pediatric population affected by brain abscesses. This observation resonates with the findings in literature [10], suggesting the need for age-stratified analyses to uncover nuanced clinical features and outcomes associated with different developmental stages.

A striking disparity emerges in the time interval between symptom onset and hospital admission, with group B demonstrating a significantly shorter duration. This highlights the critical importance of prompt medical attention, echoing recommendations from studies, emphasizing the direct impact on patient outcomes through early diagnosis and intervention [10].

The absence of statistically significant differences in perinatal characteristics between the two groups challenges established associations with neurological complications. This finding is in line with the comprehensive reviews underscoring the multifactorial nature of pediatric brain abscess etiology and the need for a broad perspective beyond perinatal considerations [1, 15].

Our exploration into the etiology of brain abscess formation elucidates post-operative neurological procedures and otopharyngeal infections as predominant causes, consistent with findings in the literature [1, 10]. Notably, the lack of association with periodontal infection challenges conventional beliefs, adding to the evolving understanding of the diverse sources contributing to brain abscess development.

Patients suspected of having a brain abscess should undergo diagnostic imaging. MRI is the preferred imaging study for brain abscesses as it is more sensitive than computed tomography (CT) scans [9]. Before initiating antibiotics in patients suspected of having a brain abscess, two sets of blood cultures should be obtained [11]. After the detection of a brain abscess and subsequent examinations, the treatment involves abscess drainage, antimicrobial therapy, pathogen-specific treatment, and, in the presence of indications [12].

Microbiological analysis underscores the prominence of Streptococcal strains, in agreement with established literature [1, 7, 13]. Successful treatment requires a combination of surgical drainage and antimicrobial therapy. The combined use of these modalities has improved the prognosis of patients with brain abscess The therapeutic regimen, comprising ceftriaxone, vancomycin, and meropenem, reflects the complex and multifaceted nature of pediatric brain abscess management [14, 10, 15]. Recurrence rates and the persistence of sequelae underscore the ongoing challenges in achieving complete resolution, warranting continued research to refine treatment strategies.

In conclusion, this study significantly advances our understanding of pediatric brain abscesses, emphasizing the necessity for a comprehensive, multidisciplinary approach. The findings underscore the pivotal role of early intervention, highlight diverse etiological factors, and advocate for continuous monitoring. Future investigations should build upon these foundations, incorporating a broader range of demographic, clinical, and microbiological parameters, to further unravel the intricate complexities of this challenging medical condition.

## Conclusion

Pediatric brain abscesses present as a rare yet significant infectious disease, with diverse demographic and clinical characteristics. This study highlights gender-specific patterns, age-related variations, and crucial time intervals between symptom onset and hospital admission. While various factors contribute to abscess formation, including post-operative procedures and infections, Streptococcal strains are prominent. Treatment involves a combination of surgical drainage and antimicrobial therapy, but recurrent cases and sequelae persist, indicating ongoing challenges. Early intervention, comprehensive imaging, and multidisciplinary approaches are crucial for optimal outcomes. Further research is needed to refine treatment protocols and understand the complexities of this condition.

## Data Availability

The datasets generated and/or analysed during the current study are not publicly available due [REASON WHY DATA ARE NOT PUBLIC] but are available from the corresponding author on reasonable request.
